# Tandem mass spectrometry in screening for inborn errors of metabolism: comprehensive bibliometric analysis

**DOI:** 10.3389/fped.2025.1463294

**Published:** 2025-02-20

**Authors:** Victoria Kononets, Gulmira Zharmakhanova, Saule Balmagambetova, Lyazzat Syrlybayeva, Gulshara Berdesheva, Zhanna Zhussupova, Aidana Tautanova, Yergen Kurmambayev

**Affiliations:** ^1^ Department of Natural Sciences, West Kazakhstan Marat Ospanov Medical University, Aktobe, Kazakhstan; ^2^ Department of Oncology, West Kazakhstan Marat Ospanov Medical University, Aktobe, Kazakhstan; ^3^ Department of General Hygiene, West Kazakhstan Marat Ospanov Medical University, Aktobe, Kazakhstan; ^4^ Department of Neonatal Pathology, Aktobe Regional Tertiary Care Center, Aktobe, Kazakhstan; ^5^ Department of Microbiology and Virology, Named After Sh.I. Sarbasova, Astana Medical University, Astana, Kazakhstan; ^6^ Consultative and Diagnostic Department, Medical Center of West Kazakhstan Marat Ospanov Medical University, Aktobe, Kazakhstan

**Keywords:** tandem mass spectrometry, inborn errors of metabolism, newborn screening, selective screening, scientometrics, bibliometric analysis, visualization

## Abstract

Tandem mass spectrometry (MS/MS) for detection of inborn errors of metabolism (IEM) is recognized as an ethical, safe, simple, and reliable screening test. Presented bibliometric analysis aims to describe the network structure of the scientific community in the study area at the level of countries, institutions, authors, papers, keywords, and sources; scientific productivity, directions, and collaboration efforts in a considered period (1991–2024, May). Using the PRISMA method, we conducted a systematic search for articles reporting using MS/MS to screen for inherited metabolic disorders and inborn errors of metabolism collected from the Web of Science Core Collection (WoSCC). A total of 677 articles out of 826, by 3,714 authors, published in 245 journals, with 21,193 citations in 11,295 citing articles, with an average citation of 31.3 per article, and an H-index of 69 were retrieved from the WoSCC. The research status of MS/MS in IEM screening was identified. The most relevant current research directions and future areas of interest were revealed: “selective screening for IEM,” “new treatments for IEM,” “new disorders considered for MS/MS testing,” “ethical issues associated with newborn screening,” “new technologies that may be used for newborn screening,” and “use of a combination of MS/MS and gene sequencing”.

## 1 Introduction

Tandem mass spectrometry (MS/MS) as a tool for detection of inborn errors of metabolism (IEM) was introduced in the 90 s (1–3) and recognized as an ethical, safe, simple, and reliable screening test (4, 5). In the same decade, tandem mass spectrometry protocols for newborn screening were developed in the USA (6–8). In the 1990 s, MS/MS made it possible to detect more than 30 inborn errors in the metabolism of amino acids, fatty acids, and other organic acids (2). Further, the number of metabolites analyzed in one cycle consecutively increased (7, 9). Over the subsequent decade, laboratories testing for metabolic disorders have implemented tandem mass spectrometry into their newborn screening programs (10–17). Expanded newborn screening (ENBS) using MS/MS has become a mandatory public health strategy in most countries (18). However, the number of diseases that can be simultaneously assessed using the same mass spectrometric technique is limited. In addition, the sensitivity and specificity of the multiplex method for different metabolites and the stability of these metabolites are not the same, which provides an advantage in diagnosing some diseases over others (19).

IEM constitutes a group of phenotypically and genotypically heterogeneous metabolic disorders caused by gene mutations encoding metabolic pathway enzymes or receptors. Deficiency or changes in the activity of essential enzymes or other proteins in intermediate metabolic pathways lead to the accumulation or deficiency of corresponding metabolites in cells or body fluids, manifesting in a wide range of diseases with clinical heterogeneity, complicating their diagnosis (18). While IEMs are generally considered hereditary disorders, they can sometimes arise from “*de novo*” mutations, meaning a new genetic change that occurs in the affected individual and is not inherited from their parents; therefore, not all cases are strictly hereditary in the sense of being passed down through generations, but can still be classified as “inborn errors of metabolism” due to the genetic origin of the condition.

IEMs are classified, considering the biochemical nature of the metabolites accumulated in each disease (20). Collectively, they account for more than a thousand individual genetic disorders, resulting in a significant social and financial burden overwhelming families, communities, and health authorities worldwide (19).

Although these disorders are rare, they are collectively numerous (21). There are population differences in the incidence of IEM (22–24). Many IEMs do not have specific clinical signs and are difficult to diagnose using clinical manifestations or routine laboratory tests alone (22). IEM typically results in irreversible neurological and psychological impairment and/or disability or death in affected children. Early diagnosis of IEM can significantly reduce the risk of death and may prevent long-term neurological complications (25–27).

Many genetic diseases, especially inborn errors of metabolism, are rare, so developing a newborn screening test for every disease is impractical. This obstacle was overcome through MS/MS technology (28). MS/MS is more sensitive, specific, reliable, and comprehensive than traditional assays. The outdated classical screening methods of one test, one metabolite, and one disease were replaced by a single test, many metabolites, and many diseases approach, first in the USA, Canada, Australia, and European countries (late 20th—early 21st century), then in some Eastern countries. MS/MS also facilitates adding new disorders to newborn screening panels (6, 13, 14). The advantages of this detection system are speed, the ability to analyze many different compounds in a single assay, and minimal requirement for assay auxiliary reagents (2). The sensitivity and specificity of this method can reach 99% and 99.995%, respectively, for most amino acid disorders, organic acidemias, and fatty acid oxidation defects (18).

MS/MS opened up the concept of multiple metabolite analysis to detect various metabolic disorders in a single analytical run. Using several analytes to detect biochemical disorders allows for constructing a metabolic profile (13, 29, 30). The adverse consequences of false-positive results are negligible regarding the health-economic benefits provided by ENBS and can be minimized through increased education, improved communication, and enhanced technology (18).

The MS/MS technique involves two mass spectrometric analyses performed sequentially with a fragmentation step in between (13). Despite the apparent simplicity of MS/MS, practical implementation and data interpretation can be complex, especially when analyzing complex mixtures or performing detailed structural elucidation. For example, MS/MS analysis for some lysosomal diseases requires additional efforts in the form of supplementary specialized equipment (5).

Screening using tandem mass spectrometry diagnoses more IEM cases than classical clinical screening (31). Thus, according to Wilcken et al., in a cohort of newborns examined using MS/MS, the prevalence of congenital errors was almost two times higher than in four previous four-year cohorts using clinical screening methods (15).

Typically, diagnosis of IEM using MS/MS involves the use of a series of confirmatory tests when IEM is suspected. For this purpose, particular guidelines have been developed (32). The ENBS program usually uses a two-tier system, classifying results as “borderline” or “diagnostic.” Infants with an initial borderline result are rescreened. Infants with diagnostic or two borderline results are referred for confirmatory testing (3). Avoiding false negatives with specific biomarkers and reducing false positives with second-tier tests is fundamental to a successful NBS program (33). First-stage screening can be performed using various variants of MS/MS, both LC-MS/MS and FIA-MS/MS (flow injection analysis-tandem mass spectrometry). First-stage NBS using a FIA-MS/MS can have high presumptive positive rates, often due to isomeric/isobaric compounds or poor biomarker specificity. These presumptive positive samples can be analyzed by second-stage screening assays using separations such as LC-MS/MS or ultraperformance liquid chromatography-tandem mass spectrometry (UPLC-MS/MS) (34). This increases the test's specificity and dramatically reduces the number of false positive results. Second-tier tests performed with LC-MS/MS are also multiplexed, simplifying workflows and allowing for efficient use of public health resources (35, 36). Next-generation sequencing (NGS) is now included in confirmatory testing in many countries (37, 38). Genomic DNA isolated from dried blood spots can be used for NGS, providing reliable sequencing results. NGS can serve as a secondary diagnostic test for NBS (39).

ENBS entails many interrelated variables that must be carefully assessed and optimized. More reports worldwide are needed to comprehensively evaluate various populations’ possible benefits, harms, and costs (40). The impact of the programs has been assessed in terms of screening effectiveness, costs, and clinical outcome (2, 14, 41, 42). It was found that screening of additional IEMs using MS/MS does not increase the cost of the program (43). Screening 23 additional MS/MS-based inborn errors of metabolism was found to approximately double their detection rate compared with conventional methods used in Germany (44). The introduction of MS/MS technology has significantly increased the detection of inherited metabolic disorders, including those not previously covered, with predictable improvements in outcomes for some disorders (45, 46). Pilot financial data comparing late diagnosis of treatable IEM with early diagnosis using MS/MS and subsequent treatment suggested that expanded screening with MS/MS would result in reduced morbidity and significant savings in chronic disease and critical care annual costs (47).

According to researchers, MS/MS in IEM screening allows the diagnosis and treatment of diseases before the onset of symptoms and thus represents a preventive medicine strategy (40, 44, 48). Cost-effectiveness studies have confirmed that the savings achieved through expanded NBS programs significantly exceed the costs of their implementation (18). Screening with tandem mass spectrometry has been found to provide better long-term outcomes for patients aged six years, with fewer deaths and fewer clinically significant impairments (41).

One of the considerable challenges in neonatal screening today is differentiating the disorders that would benefit most from ENBS using MS/MS, allowing screening programs to be adjusted accordingly (49, 50).

Evaluating the cost-effectiveness of MS/MS for neonatal screening in low and middle-income countries (LMICs) is particularly important. Khneisser et al. assessed the cost-effectiveness of IEM newborn screening in Lebanon as a model for similar countries. According to Khneisser et al., it can be argued that the direct and indirect costs saved by early detection of IEM are essential enough to justify publicly funded universal screening, especially in LMICs with high consanguinity rates, as illustrated by data from Lebanon. Direct treatment costs were shown to be diminished by half, reaching an average of US$ 31,631 per case identified. This difference more than covers the cost of starting a newborn screening program (51).

Improvements in MS/MS technology are why the authors of several studies have presented the detected IEM frequency as higher than in earlier studies (52, 53).

Nowadays, reports on the current status of neonatal screening traditionally divide the world into five regions (North America, Europe, the Middle East and North Africa, Latin America, and Asia Pacific), assessing the current situation with NBS in each region and analyzing the activities undertaken in recent years (54). However, the problem with IEM screening and using MS/MS as a screening tool may vary within each region.

The application of MS/MS for IEM screening can be studied using bibliometric analysis (BA) methods. Bibliometric analysis aims to identify knowledge gaps and knowledge clusters in a research area that may require more attention from the scientific community (55). BA uses mathematical and statistical methods to evaluate the structure, growth, development, and productivity of publications related to a specific topic. Recent advances in large-scale data analysis, advanced visualization techniques, and network analysis provide well-established tools and techniques for analysis and help to understand the structure and mechanisms of the field under study. Bibliometric studies are based on the metadata of publications rather than the textual content (full text) contained within them. The purpose of bibliometric analysis is to display quantitative and aggregated analysis results by publications, authors, institutions, countries, and keywords, identifying connections and clusters between them, depending on the research question (56). A bibliometric review, using scientometric data processing methods, has advantages over a conventional literature review because it allows for identifying critical issues in the field under study and key directions for future research. As an emerging field of information science, bibliometric analysis provides a quantitative and qualitative method for identifying research trends and visually delineating boundaries in a field of study (57). Recently, BA has been used in various fields to analyze published literature. A bibliometric analysis of the state and directions of research into hereditary metabolic disorders was carried out (58). However, such an analysis was not performed regarding using the MS/MS method in IEM screening.

The presented bibliometric analysis aims to describe the network structure of the scientific community in the field of IEM screening using MS/MS at the level of countries, institutional organizations, authors, and sources; their scientific productivity, directions, and collaboration efforts in a given period (1991–May 2024).

## 2 Materials and methods

### 2.1 Data sourses and search strategies

**Figure 1:**
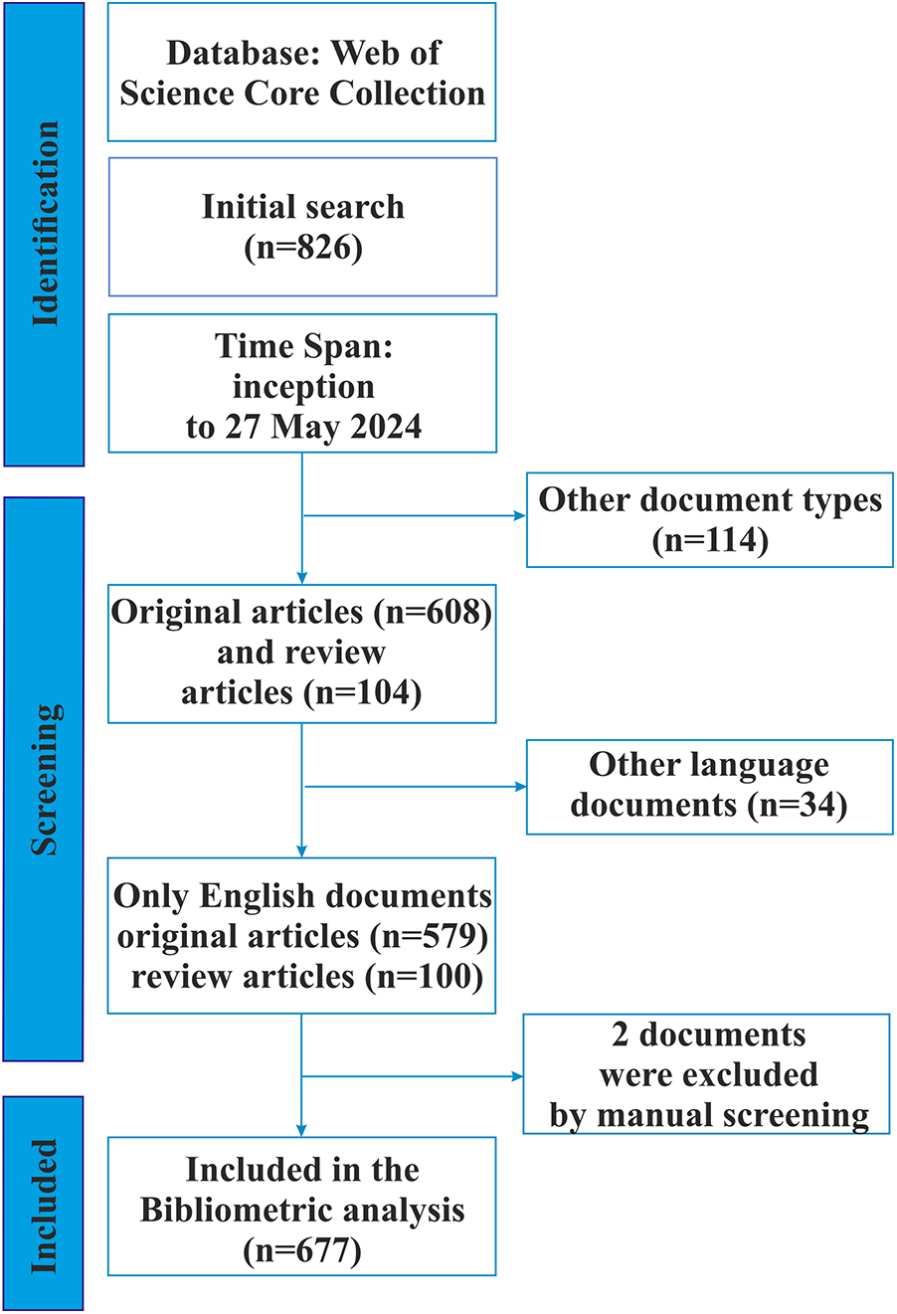
The flow chart of the screening process using PRISMA.

In late May 2024, we conducted a systematic search for articles reporting using tandem mass spectrometry to screen for inherited metabolic disorders (Figure 1). Complete records of all relevant publications were collected from the Web of Science Core Collection (WoSCC). Web of Science is traditionally considered one of the most comprehensive and authoritative database platforms and is most often used for bibliometric research (59–61).

In the current study, the search terms were as follows:

#1 (TS = (inborn errors of metabolism) OR TS = (IEM) OR TS = (inherited metabolic diseases) OR TS = (inherited metabolic disorders) OR TS = (IMD)

#2 (TS = (tandem mass spectrometry) OR TS = (MS/MS) OR TS = (LS-MS/MS)

#3 (TS = (screening) OR TS = (newborn screening) OR TS =(expanded newborn screening) OR TS = (neonatal screening) OR TS = (selective screening) OR TS = (retrospective screening)

#1 AND #2 AND #3 AND Article OR Review Article (Document Types) AND English (Languages).

As tandem mass spectrometry was first described as a potentially new method for screening inherited metabolic diseases in 1991 by Millington et al. (62), the search date was set from January 1, 1991, to May 27, 2024.

Proceeding papers, book chapters, meeting abstracts, editorial materials, early access articles, letters, and notes were excluded at the next screening stage. The language of the publications was limited to English.

During the manual screening phase, only sources regarding the use of MS/MS in IEM screening were included.

Eventually, of the 826 studies identified in the initial search, 677 met the screening criteria and were included for further analysis. The bibliometric data of the retrieved literature was downloaded as a “full record and citation” from the WoSCC database in the form of a plain text file for further analysis. Bibliometric data included publication year, title, author names, country of origin, institutional affiliation, abstract, keywords, number of citations, journal title, journal impact factor (IF), and an H-index.

### 2.2 Bibliometric analysis and visualization

Data from studies selected from the Web of Science were analyzed using the bibliometric software package RStudio (Version 2024.04.2 + 7641, PBC, Boston, MA) (http://www.bibliometrix.org; access date: 27 May 2024) (63) and Biblioshiny web applications. The software tool VOSviewer, created by the Center for Science and Technology Research at Leiden University for visualizing scientific maps, is freely available. VOSviewer (version 1.6.20) (https://www.vosviewer.com. access date: 27 May 2024) was used to build and visualize bibliometric networks created based on citations, bibliographic linkage, shared citations, or co-authorship relationships (64). Using these applications allows for the visualization of accumulated scientific knowledge in structure, distribution, and connections between them to create visualization maps that reflect progress and trends in the field under study.

We set the numerical threshold of each node (item, according to the VOSviewer terminology) of visualizations created in VOSviewer to three for country, institutional, author, and journal analysis. Thus, only elements with a number greater than three were displayed on the graphs. The size of the items (nodes) reflects the strength of each element, such as the number of citations or articles, and the distance between items (nodes) demonstrates the strength of the connection between them. The broader the communication lines between items (nodes), the stronger the cooperation.

The author analysis was carried out using indicators of fractional authorship, H-, G- and M-indices, assessing the productivity of authors over time and using Lotka's law. Fractional authorship quantifies an individual author’s contributions to a published set of papers (63).

The H-index (Hirsch index) is an author's (or journal's) number of published articles (H), each of which has been cited in other papers at least one time. The M-index is defined as H/n, where H is the H-index and *n* is the number of years since the first published paper of the scientist (journal). The G-index was introduced by Egghe in 2006 as an improvement of the h-index to measure the global citation performance of a set of articles. If this set is ranked in decreasing order of the number of citations that they received, the G-index is the (unique) largest number such that the top g articles received (together) at least g^2^ citations (63).

Lotka's law is an approximate inverse-square law, where the number of authors publishing a certain number of articles is a fixed ratio to the number of authors publishing a single article.

The number of publications was considered to identify the core of journals that contributed most to citations in the field under study, and Bradford's law was used. The Bradford's law can be used to identify “core” journals in a discipline and to focus the analysis on the core zone documents.

An analysis of the productivity of countries, organizations, authors, and journals was conducted with the determination of total (global) and local citations. It is known that the “Global citation” (GC) parameter measures the impact of documents in the whole bibliographic database. It means that any documents from the database (in our case, Web of Science) that are not included in the studied sample, represented by 677 articles, can be included in the citation. In contrast, the “Local citation” (LC) measures the number of citations a document has received from papers included in the analyzed collection. In that sense, “Local citation” more accurately measures a document's impact on the studied sample.

Bibliometric relationships, co-authorship, author countries, institutional affiliations, citations, and keywords were visualized as maps.

Top authors and institutions were ranked based on the percentage of articles they wrote. The ten countries that made the most significant contribution to writing articles in the area under consideration were also identified. Patterns of collaboration between authors, institutions, and countries were visualized.

A temporal frequency analysis of keywords was carried out. The most frequently occurring keywords and trends in using keywords in the described time interval are presented and visualized. Thematic analysis identified time trends in the selected publications.

No ethical approval was required for this study.

## 3 Results

A total of 677 articles by 3,714 authors were retrieved from the WoS Core Collection database in the timespan from 1991 to May 27, 2024, on research in the field of tandem mass spectrometry for screening hereditary metabolic disorders, published in 245 journals, with 21,193 citations in 11,295 citing articles, with an average citation of 31.3 per article, and an H-index of 69.

According to data provided by Biblioshiny, the completeness of bibliographic metadata was characterized as “Excellent” for the categories Author, Cited References, Document Type, Journal, Language, Cited References, Publication Year, Science Categories, Title, and Total Citation. Categories Affiliation, Corresponding Author, Abstract, Keywords Plus, and DOI had the status “Good,” which corresponded to a slight lack of information on them in the submitted publications, and only the Keywords category was characterized as “Poor” (missing information in articles on keywords was 22.45%.)

Web of Science classified the publications into 66 categories. The most articles were classified into the Genetics Heredity category (202 articles, 29.8%). The Pediatrics category included 145 articles (21.4%). One hundred and thirty-two articles were classified as Medicine Research Experimental (19.5%). Additionally, the top ten categories included Endocrinology Metabolism (123; 18.2%), Medical Laboratory Technology (93; 13.7%), Chemistry Analytical (76; 11.2%), Biochemical Research Methods (53; 7.8%), Biochemistry Molecular Biology (41; 6.1%,) Medicine General Internal (41; 6.1%) and Clinical Neurology (33; 3.4%) (Supplementary Figure 1).

### 3.1 Publication trends and citations

**Figure 2:**
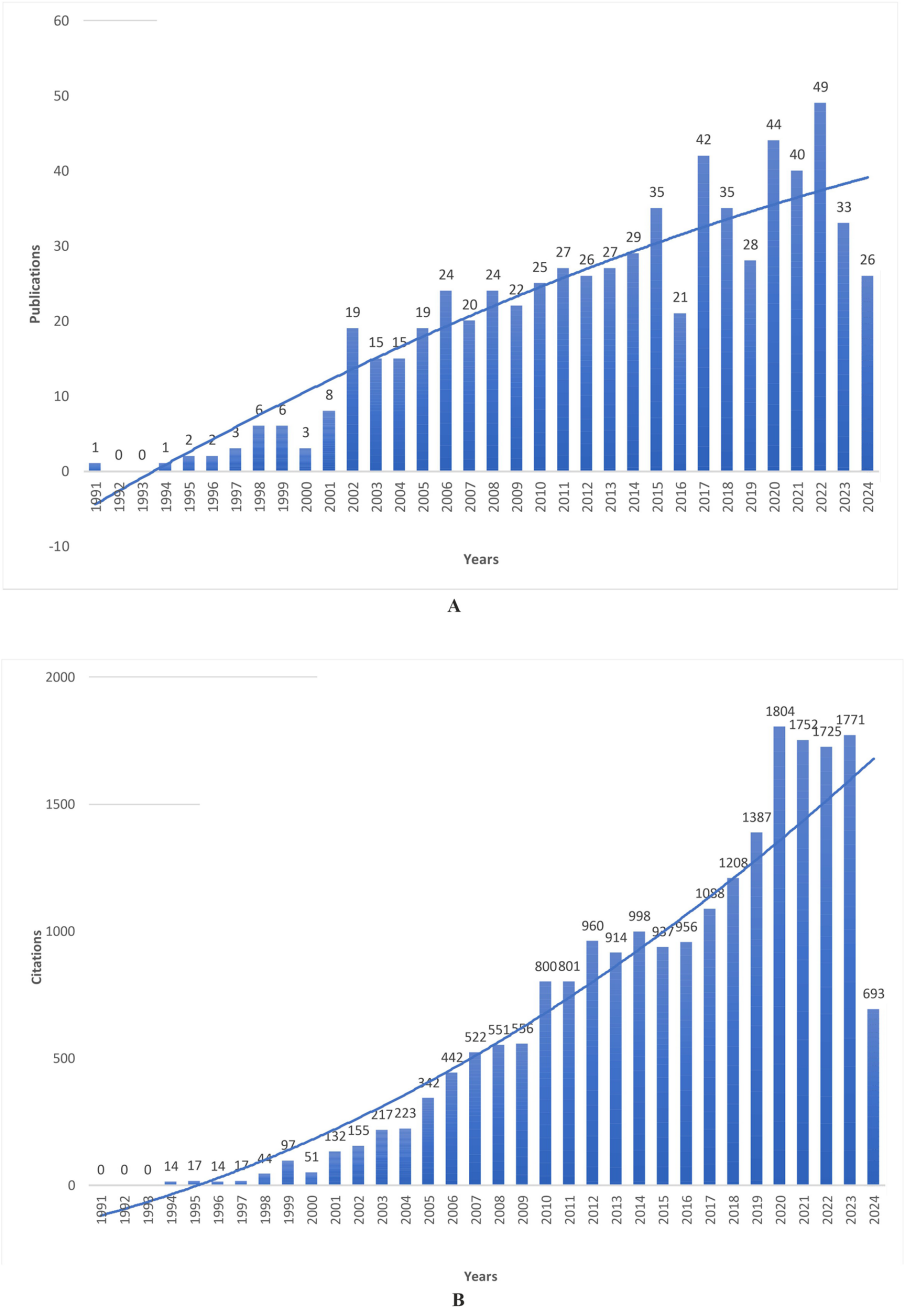
**(A,B)** Dynamics of publications **(A)** and citations **(B)** on using MS/MS for IEM screening from 1991 to May 2024.

Two stages can be distinguished when assessing the dynamics of publications on the use of MS/MS for screening hereditary metabolic disorders. The first corresponds to the last decade of the 20th century, and during this period, there was a slow increase in the number of publications and their citations. The beginning of the second stage corresponds to the start of national programs of expanded newborn screening using MS/MS in North America (13, 16, 29, 65, 66), Europe (11, 14, 44, 67), Australia (10, 15), and some Asian countries (68–70) at the beginning of the 21st century. The most significant number of articles was published in 2022 (*n* = 49); the highest number of total citations was recorded in 2020 (*n* = 1,804) (Figures 2A,B). The annual increase in the number of publications and citations, especially pronounced since the 2000s, indicates a steady interest in this topic. Although a substantial number of countries are now using MS/MS for expanded newborn screening programs for IEM, in LMICs, the use of MS/MS is associated with financial challenges (51, 71–74). This circumstance makes selective screening programs more relevant for them and explains the growing interest in the issue of using MS/MS in this area over time (23, 75–77).

### 3.2 Analysis of productivity and cooperation for countries, institutions, and authors

#### 3.2.1 Country analysis

##### 3.2.1.1 Top countries within the countries’ performance analysis

The 677 articles included in the bibliometric analysis were published in 67 different countries spanning Asia, Europe, North America, South America, Africa and Oceania (Supplementary Figure 2, Table 1), with the top 10 countries accounting for more than 72% and the top 20 countries accounting for more than 88% of the total number of articles.

**Table 1:** Countries’ scientific production.

Country	Publications	Total citations	Average article citations
USA	617	7,450	53.60
China	507	1,839	13.30
Germany	221	2,401	54.60
Italy	160	790	28.20
Japan	123	696	24.00
UK	106	934	40.60
Canada	104	373	20.70
Australia	97	1,404	70.20
Netherlands	85	637	31.90
Spain	85	412	19.60
Saudi Arabia	60	1,046	61.50
Egypt	58	94	7.80
India	58	122	6.80
South Korea	50	167	13.90
France	49	153	19.10
Austria	44	685	52.70
Brazil	42	120	17.10
Denmark	37	155	31.00
Turkey	32	105	11.70
Mexico	30	85	12.10

**Figure 3:**
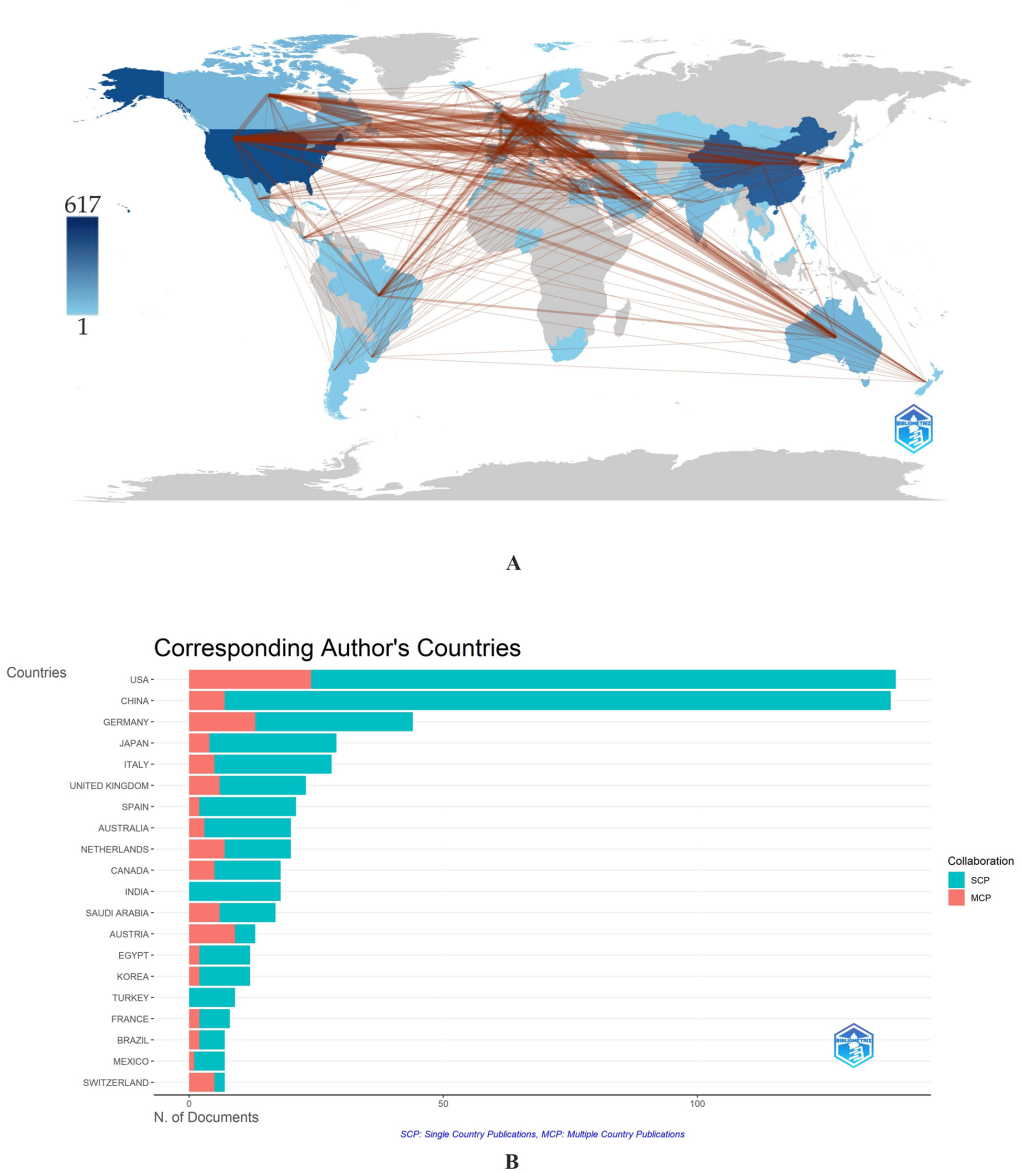
**(A,B)** Collaboration network on a world map **(A)** and Corresponding authors’ countries **(B)**. The intensity of color saturation corresponds to the increasing number of articles within each country. *MCP—Multiple Countries Publication; SCP—Single Country Publication. The red color indicates a higher level of cooperation; the broader the line of communication, the higher the level of collaboration between countries.

It is worth noting that the R-studio software's “Biblioshiny” web application offers two options for determining the inclusion of contributing countries: counting the total number of authors from different countries and assessing the countries of the corresponding authors. In the first case, the same publication is counted several times if an international team of authors wrote it. Obviously, the second way seems more optimal because each article, in this case, is counted once; besides, the number of authors in the article varies and does not accurately reflect the country's productivity. In this paper, the content of Table 1 is an expression of the first approach. In contrast, Figure 3B refers to the second approach, as it considers only the corresponding author's country when determining the inclusion of countries in the article's writing.

Research into the use of MS/MS in IEM screening is mainly concentrated in high-income countries (Table 1). The US ranks first in the number of articles with 617 (21.22%), followed by China (507; 17.43%) and Germany (221; 7.60%).

The most frequently mentioned country was the United States (7,450,) followed by Germany (2,401) and China (1,839) (Table 1). However, the distribution of positions based on average article citations places the US in 4th position (53.6), with the top three occupied by Australia (70.2), Saudi Arabia (61.5), and Germany (54.6) (Table 1).

When assessing the publishing activity of authors from different countries between 1991 and 2024, it is noticeable that publishing activity began to increase in the early 2000s (Supplementary Figure 2). In the decade preceding 2000, a few works were presented primarily by authors from the USA and the United Kingdom (UK). The number of publications increased sharply when the era of national neonatal screening programs using MS/MS began. The leading countries in publications and citations include the USA, China, Germany, Italy, Japan, the UK, Canada, Australia, Netherlands, Spain, and Saudi Arabia (Table 1, Supplementary Figure 2).

##### 3.2.1.2 Maps of cooperation between countries

The issue of IEM early diagnosis is of high importance for public health in all countries. Authors from 67 countries demonstrate global collaboration trends in research on using tandem mass spectrometry for IEM screening (Figures 3A,B).

The Multiple Countries Publication (MCP) is an essential indicator of international cooperation. MCP indicates the number of documents with at least one co-author from another country for each country, thus measuring the intensity of collaboration between countries. The ratio between SCP (Single Country Publication) and MCP determines the MCP Ratio, a high value that indicates intensive collaboration between authors from different countries when writing articles. Thus, the Netherlands, Germany, Saudi Arabia, Canada, Switzerland, and Austria have high MCP and MCP Ratios, according to Figure 3B. In contrast, the MCP for Turkey and India is zero, reflecting their low levels of international collaborative activity.

The collaboration map created in VOSviewer visualizes the cooperation situation between the leading countries. Out of 67 countries, VOSviewer showed 41 countries with more than three publications (Supplementary Figures 3A,B). However, five countries (Thailand, Portugal, Greece, Iran, and Slovenia) had no connections with others and were not reflected on the visualization map. Thus, the final number of countries included in the analysis was 36.

The USA, Germany, Great Britain, Netherlands, Australia, and Canada have the highest intensity of cooperation. Maps of collaboration between countries represent 7 clusters, whose centers are the USA (purple), Netherlands (orange), Germany (red), Denmark (yellow), Canada (dark blue), Australia (blue), Great Britain (green) (Supplementary Figure 3A). The highest degree of territorial segregation is noted in the red and yellow clusters, which include only European countries, with a single exception. In contrast, the remaining clusters include countries from around the world. Figure 3B, apart from presenting the number of documents from collaborating countries, reflects their average citations. This parameter is highest in Australia, Canada, and Saudi Arabia despite the relatively small number of published articles.

In the analysis of collaboration between countries with more than three publications (Supplementary Figure 3A), clusters are identified by color. Item (node) sizes correspond to the number of publications, and the distance between them indicates the strength of the co-authorship relationship. In Supplementary Figure 3B, the item (nodes) sizes correspond to the number of publications, the color of the items (nodes) varies from dark blue to yellow according to the average article citations parameter, and the distance between them shows the strength of the connection according to the “Co-authorship” parameter.

#### 3.2.2 Institutional analysis

##### 3.2.2.1 Leading institutions

Eight hundred twenty-six institutions worldwide participated in IEM screening studies using MS/MS. Eighty-seven institutions published ten or more articles, thirty-eight published 15 or more articles, and twenty-two leading institutions published more than 20.

As shown in Supplementary Figure 4A, Egyptian Knowledge Bank (EKB) (n 54; 7.98%) has the highest number of publications, followed by Ruprecht Karls University Heidelberg (n 35; 5.17%), while the University Medical Center Hamburg-Eppendorf, University of Hamburg and the Mayo Clinic (n 32; 4.73%) rank third. Also in the top 10 most productive institutions are the University of Sydney (30), the University of Ottawa (29), Shanghai Jiao Tong University (27), the University of Amsterdam (27), and the University of Hong Kong (26). Of the top 10 leading organizations, the Egyptian Knowledge Bank (EKB) is the most productive, even though the country ranks 12th in productivity ranking (Table 1). Most of the publications of Egyptian authors (54 out of 58) are affiliated with the Egyptian Knowledge Bank, the largest Egyptian scientific online library. It accumulates scientific products from all Egyptian universities and is represented as a scientific organization in WoS.

An analysis of the scientific output distribution in the period under consideration shows that a surge of interest in using MS/MS in IEM screening in different organizations occurred at different times. Most leading organizations were involved in neonatal screening programs in the early to mid-2000s and 2010s, and some, such as the Egyptian Knowledge Bank (EKB) (Supplementary Figure 4B), have been doing so in the last few years.

##### 3.2.2.2 Maps of institutions cooperation

**Figure 4:**
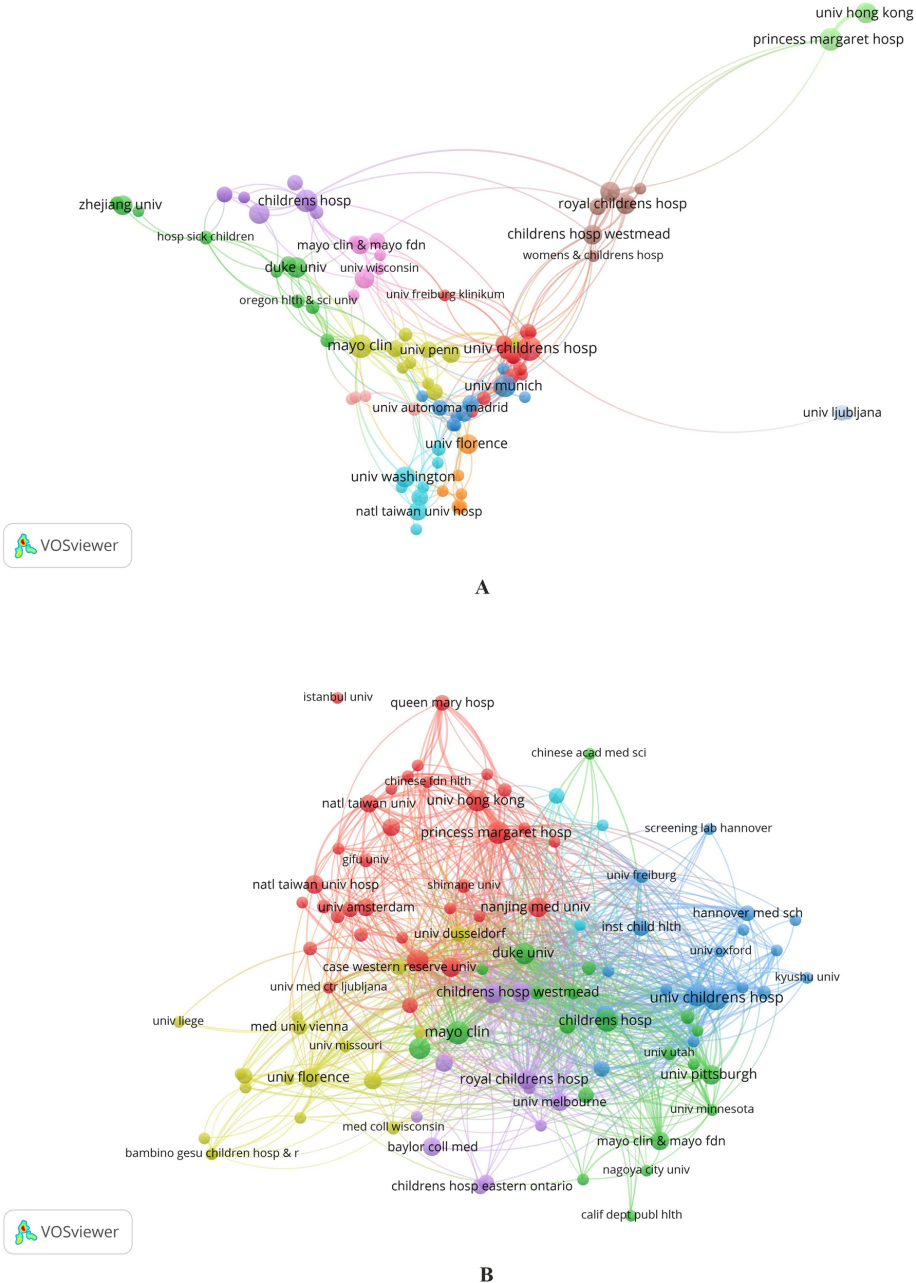
**(A,B)** Maps of institutions’ cooperation. Collaboration between organizations in terms of co-authorship by clusters **(A)** Connection between the formed 6 clusters of organizations based on the “Citation” indicator **(B)**.

The co-authorship collaboration map created in VOSviewer analyzes collaborations between 827 institutions belonging to 67 countries. The visualization map initially included 104 institutions with more than three publications. However, 13 were not included in the final visualization map because they had no connections with other organizations. Thus, only 91 organizations are represented on the cooperation map (Figure 4A,B).

Maps of collaboration between organizations in the field of co-authorship represent 12 clusters (Figure 4A). Institutions in Europe and the USA have the highest intensity of cooperation. Of particular note is that Egyptian institutions, which have the most significant number of publications in the field under consideration, are not included in the cooperation map.

In the analysis of collaboration between institutions with more than three publications (Figure 4A), clusters are identified by color, the size of the items (nodes) corresponds to the number of publications, and the distance between them shows the strength of the relationship regarding co-authorship. In Figure 4B, the sizes of the items correspond to the number of documents, and the distance between them shows the strength of the connection according to the “Citation” parameter.

Figure 4B shows the strength of the connection between the formed 6 clusters of organizations based on the “Citation” indicator. There is a tendency to include mainly territorially close institutions in clusters; for example, blue and cyan clusters mainly include organizations in Northern Europe, yellow—in Southern Europe, red—in Southeast Asia, purple—in North America, and green—in Australia. However, within each cluster, there are also quite geographically distant institutions, demonstrating trends in international cooperation between organizations and mutual interest in publishing articles.

#### 3.2.3 Analysis by authors

##### 3.2.3.1 Authors’ productivity analysis

Three thousand seven hundred fourteen authors have published literature on using MS/MS in IEM screening, and ten have published more than ten articles. Twenty-nine authors submitted papers written without co-authors. The average number of authors per document was 7.99. International author collaborations amounted to 18.46%.

Collectively, the top 10 authors published 131 papers, representing 19.4% of all publications in the field. The number of publications and citation rates of the ten most productive authors are summarized in Table 2.

**Table 2:** Most productive authors.

Rank	Author	Articles/% of 677	Articles fractio nalized	Total number of citations	H- index	G- index	M- index	Publishing since
1	Hoffmann G.F.	20 (2.95%)	2.23	1,558	18	20	0.783	2002
2	Wang Y.	15 (2.22%)	1.99	190	7	13	0.389	2007
3	Matern D.	14 (2.07%)	2.72	904	11	14	0.478	2002
4	Vockley J.	14 (2.07%)	1.36	573	11	14	0.478	2002
5	Chace D.H.	12 (1.77%)	4.65	1,103	11	12	0.324	1991
6	Gu X.F.	12 (1.77%)	1.32	438	7	12	0.389	2007
7	Wilcken B.	11 (1.63%)	1.74	1,635	11	11	0.478	2002
8	Kölker S.	11 (1.63%)	0.98	769	10	11	0.435	2002
9	Mak C.M.	11 (1.63%)	1.23	204	6	11	0.429	2011
10	Zhang Y.	11 (1.63%)	1.11	123	6	11	0.316	2006

The three authors with the most publications were Georg F. Hoffmann (20), Yuqi Wang (15) and Dietrich Matern (14). No less than the number of articles, the author's publication activity is characterized by the indicator of fractional frequency or fractionalized number of authored documents (Table 2). Fractional frequency by Donald H. Chace is several times higher than the corresponding indicator of other top ten authors.

The indicators of total (Table 2) and local citations (Supplementary Figure 5A) are also essential in assessing the author's productivity. Three authors in the current selection had a total citation score above 1,000. The most frequently cited authors were Bridget Wilcken (1,635), Georg F. Hoffmann (1,558), and Donald H. Chace (1,103) (Table 2). In addition, the author's local impact by H, G, and M indexes were analyzed (Table 2). The H- and G- indexes reflect authors’ productivity and citation rates. In contrast, the M-index demonstrates the relationship between the productivity and citation of the author and the number of years of intensive activity.

The productivity of the top 10 authors throughout their activity was also analyzed using the R package bibliometric matrix shown in Supplementary Figure 5B. The graph represents periods of authors’ careers in the field described and allows for evaluating the productivity and citation of authors from the top ten.

The authors’ productivity in studying the use of MS/MS in IEM screening is described by Lotka's law. In our case, the number of authors who wrote one article on using MS/MS in IEM screening is 2,880, or 77.5% of the total authors (Supplementary Figure 5C). The minority of authors who published many articles (in this case, three documents) is 4.1%.

##### 3.2.3.2 Author collaboration maps

**Figure 5:**
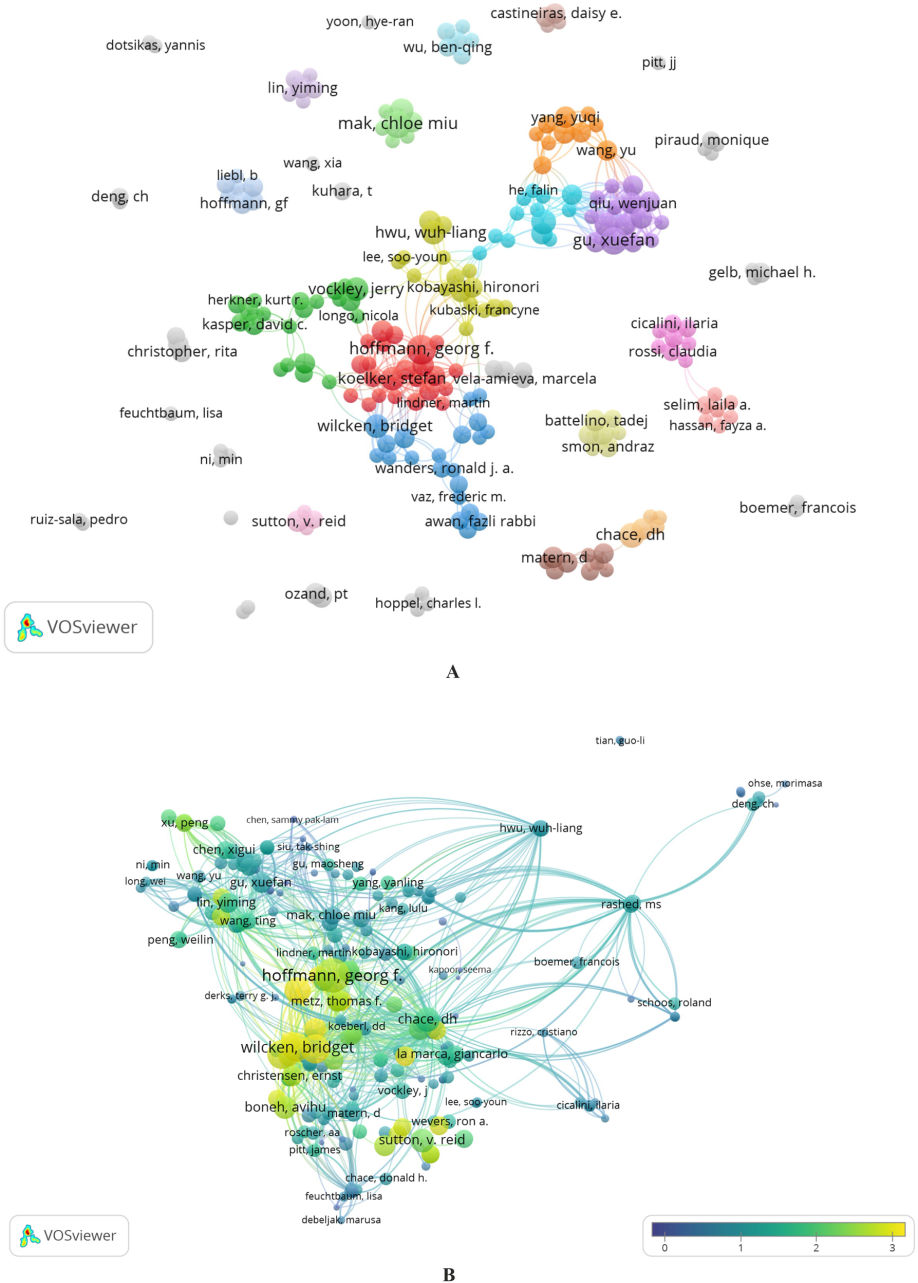
**(A,B)** Distribution of co-authors’ collaborations. Cluster analysis of collaboration between authors with more than three publications **(A)**. Collaboration between authors by the “Citation” dimension created in VOSviewer **(B)**.

Figure 5A shows a visualization of collaboration between 231 authors who have published at least three articles investigating the use of MS/MS in IEM screening. These authors formed 37 clusters. Collaboration between authors is reflected in the number of connections within clusters but is minimal between separate clusters. This is easily explained if we remember that there are few authors (amounting to about 4%) with more than four articles and, thus, more significant collaboration opportunities (Supplementary Figure 5C). We found six authors who were not co-authors of any studies and worked alone.

Figure 5B visualizes a map of collaboration between authors by the “Citation” dimension created in VOSviewer. An Overlay Visualization layer was applied to evaluate the weights and scores of the “Citation among Authors” parameter. The normalized citation parameter is used to represent scores. The normalized number of citations for a document equals the number divided by the average number of citations of all documents published in one year and included in the data provided by VOSviewer. Normalization corrects for the fact that older documents had more time to be cited than more recent documents (78).

In Figure 5A, clusters are identified by color, the size of the items (nodes) corresponds to the number of publications, and the distance between them shows the strength of the connection according to the “Co-authorship” parameter. In Figure 5B, the sizes of the nodes correspond to the number of citations of the author; the color range of the node from dark blue to yellow shows the score for the normalized citation parameter.

### 3.3 Analysis by journals

#### 3.3.1 Prominent journals

All articles (677) were published in 245 journals belonging to 91 publishing houses. The top three publishers that issued more than half of the articles reviewed were Elsevier (195 articles; 28.80%), Springer Nature (96; 14.18%), and Wiley (84; 12.40%).

Core sources by Bradford's law and sources’ productivity over time are displayed in Supplementary Figures 6A,B.

According to Bradford's Law, journals are ranked in descending order of articles on a topic, forming sequential zones. In line with Bradford's Law, the first zone included nine journals that published articles on the use of MS/MS in IEM screening that can be considered the best choice for researchers in the field.

The performance of the nine leading journals in the field of publications on the role of MS/MS in screening for IEM, defined according to Bradford's Law, over the period 1991–2023, is presented in Supplementary Figure 6B. This graph reflects the steady increase in interest to the designated problem in the scientific world. The increase in growth since 2011 is apparently due to the successful implementation of national neonatal screening programs for IEM using MS/MS in several countries and the publication of research results. Publications on the use of MS/MS in IEM screening have been published in the Journal of Pediatric Endocrinology & Metabolism since 2013, in Frontiers in Genetics since 2018, and in the International Journal of Neonatal Screening since 2020. All of them are currently included in the Core Sources defined according to the Bradford Law.

Table 3 shows the top 15 journals ranked by number of publications on using MS/MS for IEM screening.

**Table 3:** Most relevant journals.

Rank	Journal	*N* of Publications/% of 677	Total *N* of citations	H-index	G-index	M-index	Publishing since
1	Molecular Genetics and Metabolism	51 (7.53)	1,575	26	39	1.083	2001
2	Journal of Inherited Metabolic Disease	46 (6.79)	2,320	29	46	1.074	1998
3	Clinica Chimica Acta	30 (4.43)	800	16	28	0.667	2001
4	International Journal of Neonatal Screening	21 (3.10)	168	7	12	1.4	2020
5	Clinical Chemistry	20 (2.95)	1,976	17	20	0.607	1997
6	Clinical Biochemistry	20 (2.95)	581	12	20	0.48	2000
7	Journal of Pediatric Endocrinology & Metabolism	18 (2.66)	133	7	10	0.467	2010
8	Orphanet Journal of Rare Diseases	17 (2.51)	419	9	17	0.643	2011
9	Frontiers in Genetics	14 (2.07)	126	6	11	0.857	2018
10	Pediatrics	14 (2.07)	1,109	13	14	0.542	2001
11	Rapid Communications in Mass Spectrometry	12 (1.77)	384	11	12	0.355	1994
12	Journal of Chromatography B-Analytical Technologies in the Biomedical and Life Sciences	10 (1.48)	330	8	10	0.348	2002
13	Journal of Medical Screening	9 (1.33)	137	7	9	0.636	2014
14	Analytical Chemistry	8 (1.18)	290	8	8	0.5	2009
15	Analytical and Bioanalytical Chemistry	7 (1.03)	284	6	7	0.3	2005

To better assess the significance of these journals in studying the problem of using MS/MS in IEM screening, Table 3 also includes indicators of total citations, H-index, G-index, and M-index. Based on the totality of data, the most influential journal in this area is Molecular Genetics and Metabolism (articles 51; total citation 1,575; H-index 26). Regarding total citations, the Journal of Molecular Genetics and Metabolism is inferior to those occupying 2nd and 5th places in the top list.

In addition to Molecular Genetics and Metabolism, the top five journals that published the most significant number of studies included the Journal of Inherited Metabolic Disease (46 publications; 2,320 citations), Clinica Chimica Acta (30; 800), International Journal of Neonatal Screening (21; 168) and Clinical Chemistry (20; 1,976.) The total citation rate is the highest in Clinical Chemistry and the Journal of Inherited Metabolic Disease. However, these journals also have a more extended period of publication, from 1997 to 1998, respectively, which increases the total citation rate for published articles. On the contrary, the International Journal of Neonatal Screening, with a relatively low total citation index (168) has the highest M-index (1.4), which is due to high productivity and citation rates over a short period of activity (since 2020) comparing to other journals.

#### 3.3.2 Journal collaboration maps

Supplementary Figure 7A visualizes a map of collaborations between journals that have published at least three articles investigating the use of MS/MS in IEM screening. Thirty-two journals formed 6 clusters identified by color. The item (node) size corresponds to the number of publications. It is determined by the leading journals in each cluster. The distance between journals shows the strength of the connection according to the “Citation” parameter. The presence in the same cluster also demonstrates the collaboration between them in the field of citations.

Supplementary Figure 7B shows interactions between journals based on citations of published papers on the topic under consideration. Similar to Supplementary Figure 7A, the distance between journals serves as an estimate of the relationship between them based on citations. However, this visualization also allows for evaluating the level of total citation of sources by the size of items (nodes) and the parameter of normalized citation by their color. The nodes’ sizes correspond to the journal's total number of citations of the journal; the color range of the node from dark blue to yellow shows the score for the “Normalized citation” parameter.

Co-citation relationships between journals are also visualized in Supplementary Figure 7C, which shows how 109 journals form 4 clusters. The sizes of the nodes correspond to the number of co-citation references, and the distance between them shows the strength of the connection according to the co-citation parameter. The minimum number of co-citations was set to 30. The Journal of Inherited Metabolic Disease ranks first out of 245 co-cited journals (2,192 co-citations), followed by Clinical Chemistry (1,378) and Molecular Genetics and Metabolism (1,258).

### 3.4 Analysis by papers

#### 3.4.1 Highly cited papers. Analysis of citation

Overall, 43 documents out of 677 analyzed have more than 100 citations. Supplementary Table S1 includes the 15 most cited articles on MS/MS for IEM screening. The total number of citations for these 15 articles ranged from 187 to 495. The total citations are visualized in Supplementary Figures 8A–D section A. The 15 most cited articles were published in 11 journals. Although the presented 15 articles are ranked by total citation, the “Local citation” indicator, the “Total citation,” and the LC/TC Ratio associated with these parameters are of great significance (Supplementary Table 1, Figure 8B).

For instance, the paper “Screening newborns for inborn errors of metabolism by tandem mass spectrometry,” which has the highest global citation rate, is also ranked first in the local citation. However, some of the 15 most cited articles have low local citations. Among them, “Dried blood spot sampling in combination with LC-MS/MS for quantitative analysis of small molecules” (7), “Diagnosis and management of glutaric aciduria type I—revised recommendations” (8), “Neonatal screening for lysosomal storage disorders: feasibility and incidence from a national study in Austria” (8), and “Disorders of mitochondrial long-chain fatty acid oxidation and the carnitine shuttle” (2). Supplementary Figure 8B presents the 15 publications with the highest “Local citation” values, and 10 of them are included in Supplementary Table 1 as articles with the highest “Total citation” scores. All these articles are cited in the present paper (column “References”).

#### 3.4.2 Analysis of co-citation

When constructing visualization maps in VOSviewer, we used methods for assessing co-citation and bibliographic coupling, allowing for building significantly denser networks than networks of co-authorship or direct citation. Using co-citation maps and bibliographic coupling, the thematic and semantic structure of the studied topic, “Application of MS/MS in IEM screening,” can be presented more clearly. The co-citation map (Supplementary Figure 8C) shows sources that have been co-cited more than 25 times. The sample of 677 articles contains 46 such papers combined into 3 clusters on the visualization map.

In the co-citation map of publications from the study sample, presented in Supplementary Figure 8C, the co-citation indicator is visualized as the frequency with which other papers cite two articles together. The frequency of co-citation determines the semantic proximity of publications.

On the bibliographic coupling visualization map (Supplementary Figure 8D) the proximity of two publications is determined by the citation of the same sources in them. Similar sets of references in articles increase the strength of the connection between them and indicate proximity within the topic being studied.

### 3.5 Analysis of keywords

#### 3.5.1 Authors’ and additional (plus) keywords

The authors’ and additional keywords have been studied using Biblioshiny and VOSviewer.

This study analyzed 1,344 keywords. The top 20 author keywords and the top 20 additional keywords with the highest frequency of matches are displayed in Supplementary Figures 9A–D, sections A, and B.

Among the authors’ keywords, “newborn screening” (209) has the highest frequency of occurrence, followed by “tandem mass spectrometry” (149) and “inborn errors of metabolism” (137). Among the additional keywords, “tandem mass spectrometry” (295) has the highest frequency of occurrence, followed by “inborn errors” (272) and “metabolism” (197). The cumulative frequencies of occurrence of the authors’ and additional keywords are presented in sections A and B in Supplementary Figure 9. In contrast, sections C and D show a chronological analysis of the most significant keywords. From 2005 to 2006, the cumulative frequencies of use of the most significant authors’ and additional keywords incrementally increased.

Trending topics of keywords and the evolution of the authors’ keywords are presented in Supplementary Figures 10A,B. The peak citations of the abovementioned keywords occurred between 2011 and 2015 (Supplementary Figure 10, section A), corresponding to when national neonatal screening programs for IEM using MS/MS were actively conducted and reported in most European countries, the USA, Australia, and some Asian countries. However, screening programs have only been successfully implemented in high-income countries. Financial problems associated with expensive neonatal screening programs made their implementation problematic in LMICs, necessitating the search to solve these challenges by implementing selective screening programs for IEM. As such, the current priority is to obtain screening results for IEM in developing countries, as evidenced by the fact that “developing countries” is the most commonly used term in 2023 (Supplementary Figure 10A).

#### 3.5.2 Keywords co-occurrence maps and relationships between authors, keywords, and sources

The co-occurrence of keywords was analyzed using the visual mapping program VOSviewer (Supplementary Figures 11A–D, section A). The authors’ keywords and keywords plus were analyzed together. For the 1,344 original and additional keywords found in the articles’ texts, the minimum occurrence threshold was set to 7 times, which resulted in 147 extractions. The largest items (nodes) among the seven clusters in Supplementary Figure 11A are “tandem mass-spectrometry,” “inborn errors of metabolism,” and “newborn screening,” which coincides with the topic of the presented study. After removing keywords such as “tandem mass-spectrometry,” “mass spectrometry,” “inborn errors of metabolism,” “inborn error of metabolism,” “inborn errors of metabolism (IEM),” “metabolic disorders,” “inborn errors,” “inherited metabolic diseases screening,” which could affect the analysis, 128 keywords formed seven clusters. However, the nodal keywords were “newborn screening”, “metabolism”, “disorders”, “dried blood spots”, “deficiency”, “diagnosis”, and “acylcarnitines” (Supplementary Figure 11B).

Additionally, keywords extracted from the articles’ titles and abstracts were analyzed. After removing keywords such as “tandem mass-spectrometry,” “mass spectrometry,” “inborn errors of metabolism,” “inborn error of metabolism,” “inborn errors of metabolism (IEM),” “metabolic disorders,” “inborn errors,” “inherited metabolic diseases screening,” “LS-MS/MS” out of 14,876 keywords, 252 overcome the established threshold of occurrence in 20 words. The most relevant 60% (151) were included in the final analysis (Supplementary Figure 11C).

Three-field plots were constructed in Bibliometrix to evaluate relationships between authors, keywords, and journals (Supplementary Figure 11D). The most significant number of references to trending keywords such as newborn screening (11), Tandem mass-spectrometry (7), and Inborn errors of metabolism (5) was provided by the author Wang Y. The most significant number of communications with leading journals publishing articles on the topic under consideration was noted in Wang Y. (5), Gu X.F. (6), and Chien Y.H. (8).

## 4 Discussion

The last decade of the 20th century was marked by publications proposing MS/MS as a new screening tool for inherited metabolic diseases, including neonatal screening. The first publication revealing the potential of MS/MS in the field of screening was the publication of a short report by Millington et al. in the Proceedings Papers of the 1989 Annual Meeting of the Society for the Study of Inborn Errors of Metabolism (not included in the present study) (79). The first article in the sample under review, published in 1991, analyzing diagnostic markers of genetic disorders in human blood and urine using tandem mass spectrometry, was also by Millington et al. (62). The first papers on using MS/MS in IEM screening were published in the USA. At the first stage of publications in this area, which corresponds to the 90s of the 20th century, and at the second, in the 21st century, the leadership of the United States is undeniable (Table 1, Supplementary Figure 2 and Figure 3B).

Pilot studies of MS/MS use for newborn screening were first initiated in the USA, in Pennsylvania, Ohio, North Carolina, and Louisiana in 1992–1999 (80). A pilot project using universal tandem mass spectrometry for newborn screening began in North Carolina in 1997 to determine the frequency and feasibility of screening for fatty acid oxidation disorders, organic acids, and selected amino acids (3). As of 1998, twenty-six US states used MS/MS to screen for IEM in newborns (43), and this moment corresponded to the surge in publications in 1997–1999 (Figures 2A,B). However, it is worth noting the involvement of Saudi Arabia in the use of MS/MS as a screening tool (Supplementary Figure 2 and Table 1), which was especially evident at an early stage in the 90s, owing to the publications of Rashed et al. (81–83). Involvement at the level of countries, institutional communities, and individual authors in the problem under study is undoubtedly determined by the current capabilities, including financial and technical ones, and the need to use the IEM diagnostic method. Thus, Saudi Arabia's involvement may be due to the significance of increased IEM frequency in the Middle East region due to the high degree of consanguinity and the country's financial capabilities to implement screening programs for IEM using MS/MS (77, 84, 85).

The US leadership in the field of IEM screening using MS/MS is determined by differences in approaches to the number of diseases included in the neonatal screening panel and, in general, differences in the pace of screening programs in the USA and other countries, including Europe. In Europe, initially, only certain diseases were recommended for inclusion in the neonatal screening panel for MS/MS, such as phenylketonuria (PKU), glutaric aciduria type 1 (GA1), and medium-chain acyl-coenzyme A dehydrogenase deficiency (MCAD) (12, 14, 43). In Great Britain, at the beginning of the 21st century, laboratories routinely used MS/MS to screen for phenylketonuria, and only laboratories participating in the 2-year pilot study screened for MCAD. In general, the appropriateness of MS/MS to detect other IEMs was questioned (86). In the analyzed articles’ texts, “Phenylketonuria,” “CoA dehydrogenase deficiency,” and “Medium-chain acyl-CoA” were among the most frequently used original (Supplementary Figure 9A) and additional keywords (Supplementary Figures 9B,D). In Germany, the number of IEMs subject to screening was limited compared to the US during that period. On the contrary, an extensive range of IEM was recommended in the USA, including rare ones or those with unproven clinical significance (66). In the USA, MS/MS has been widely used for newborn screening for up to 55 abnormal biochemical conditions, while in Germany, the UK, and Switzerland, for the limited detection of only a few diseases (9, 87).

Australia should also be considered a leader in the use of MS/MS in IEM screening (Supplementary Figure 2, Figure 3B and Table 1), which is especially significant at the first stage of the MS/MS introduction in neonatal screening in the 90s of the 20th century (Supplementary Figure 2). The University of Sydney tops the list of significant affiliations (Supplementary Figures 4A,B). Australia's leading position is mainly due to the high citation rates of B. Wilcken's papers (Table 2), in particular, Wilcken et al. (15), which ranks first in the list of the 15 most cited articles on the topic under study and has the highest Total Citation index 495, average citations per year (TC per Year) 22.5, and Local Citations 150 (Supplementary Table 1).

MS/MS technology has offered a new vision for newborn screening programs, allowing the detection of dozens of metabolic abnormalities in a single test from a single small spot of dried blood. In the first decade of the 21st century, after several million newborns worldwide were screened and more than 500 cases of inherited metabolic diseases were identified, screening newborns with MS/MS has proven its advantages as a clinical screening technology (13, 29). It marked a new phase in the use of MS/MS for IEM screening, and the early 2000s saw a sharp rise in the number of studies on this topic (Figures 2A,B). Undoubtedly, a significant contribution, especially at the initial stage, to the promotion of the use of MS/MS in IEM screening was made by publications in the field of clinical and analytical chemistry and clinical biochemistry (Supplementary Figure 1) (1, 30, 65, 88), published in the journals Clinica Chimica Acta, Clinical Chemistry, Clinical Biochemistry, Analytical Chemistry, Journal of Chromatography B, Analytical and Bioanalytical Chemistry, included in the 15 most relevant journals that published papers on the topic under study (Table 3) and in 8 journals that formed the first zone according to Bradford's Law (Supplementary Figure 6A).

Newborn screening deals with rare diseases, and its benefits cannot be easily demonstrated without extensive studies (89). The adoption of national neonatal screening programs has resulted in the publication of study results (10, 15, 16, 31, 68, 90–94), case reports (95–97), systematic reviews and meta-analyses (98, 99), and expert reports and opinions (43, 100–102), development of methodological recommendations (103–105) and clinical guidelines (32, 103). Many papers on the topic under consideration have high rates of total and local citations (Supplementary Table 1, Figures 8A,B). Interest in using MS/MS for IEM screening continues to increase, with publications and citations peaking in 2020–2022 (Figures 2A,B).

National newborn screening programs based on MS/MS and other newborn screening technologies show significant variation in the screening panel's number and types of diseases (31, 40). Pilot neonatal screening programs using MS/MS have been launched in Europe (2, 31, 106, 107), Australia (10, 15), Asia—Japan (68), Korea (69), China (94, 108), and Taiwan (93)—already at the end of the 20th-beginning of the 21st century.

The implementation of MS/MS in neonatal screening programs in a significant part of developed countries was completed in the 2010s in Europe—in Germany (40, 109), Austria (104, 107, 110), Italy (46, 111, 112), Spain (113), Portugal (114), Denmark (31), in Asia—Taiwan (93, 115) and Singapore (116).

Currently, neonatal screening programs for MS/MS are actively implemented in European countries: Germany (117, 118), Slovenia (25, 38, 119), Italy (120, 121), Spain (122), as well as in China (52, 53, 123–129) (Supplementary Figure 2). The cited publications estimate the incidence of various IEMs in newborns and the geographic distribution of these disorders. The incidence varies within different racial and ethnic groups, with the predominance of one or another IEM in certain groups (25). Differences in the frequency of certain IEMs determine the inclusion of different IEMs in national screening panels (20, 23, 128, 130). In turn, it determines differences in the relevance and frequency of using different keywords in different regions, countries, and periods (Supplementary Figures 9C,D, 10A,B).

In China, the most common hereditary diseases, especially in newborns/infants, are hyperphenylalaninemia (HPA), citrin deficiency, primary carnitine deficiency (PCD), methylmalonic acidemia (MMA), and multiple-CoA dehydrogenase deficiency (MADD) (20, 124, 126). MMA has been frequently detected in Japan, China, and India. ENBS found differences in overall IEM rates across countries: 1:8,557 in Japan, 1:7,030 in Taiwan, 1:13,205 in South Korea, and 1:2,200 in Germany. Frequently detected diseases included propionic acidemia (PA) and PKU in Japan, 3-methylcrotonyl-CoA carboxylase deficiency (MCCD) and PKU in Taiwan, MCCD and citrullinemia type I citrullinemia I (CIT I) in South Korea, as well as PKU and MCAD in Germany.

Thus, the incidence rate of IEM varies among countries. Moreover, the disease spectra of inherited metabolic diseases (IMD) detected by selective screening differ from those detected by expanded newborn screening (23). The overall incidence of fatty acid oxidation disorders (FAOD) in Asians is much lower than in Caucasians. The significant prevalence and apparent benefit of ENBS for MCAD screening has only been demonstrated in countries with a high percentage of Caucasians (102). It determines the focus of individual countries, organizations, and authors on the development of diagnostics of some IEM groups relevant to them and the formation of collaborations between authors (Figures 5A,B), organizations (Figures 4A,B), and countries (Figures 3A,B and Supplementary Figures 3A,B) based on these interests.

It is noteworthy that even within the same country, the degree of formation and development of neonatal screening programs may vary. In China, the spread of MS/MS technology in neonatal screening in some regions, particularly the North (131), Midwest (94, 132), and Hong Kong (94, 133), was implemented later than in other parts of mainland China. The same could be said of the United States, as newborn screening within the US is an “states rights” “issue (3, 8, 16, 48, 62, 97, 101).

In some countries, selective screening programs for IEM using MS/MS have been implemented concurrently with expanded newborn screening. This IEM screening strategy has been actively used in China (96, 97, 134–136), Korea (69), Slovenia (25, 38, 119), India (73), Turkey (21), and Egypt (137).

It should be noted that there are some financial issues with carrying out ENBS using MS/MS, particularly in South-Eastern European countries (138). In India, economic constraints in the health care system have prevented the implementation of a full-scale enhanced neonatal screening program. Pilot studies using MS/MS to assess the prevalence of IEM were initiated in Andhra Pradesh as early as 2004 (73, 139), and selective screening for IEM in India continues to this day (140, 141). There is a high prevalence of IEMs, but more extensive studies are required to estimate their true prevalence in India. One of the problems associated with IEM screening programs in India is the lack of international collaboration in conducting research and publishing its results, as reflected by the high SCP (Single Country Publication) and zero MCP (Multiple Countries Publication) (Figure 3B) and lack of representation India on the international cooperation visualization map (Supplementary Figures 3A,B).

Many developing countries do not yet have national neonatal screening programs (72). In most developing countries, there are financial challenges to implementing expanded neonatal screening programs. Pilot programs with limited observations or selective screening programs are being implemented in these settings. Pilot programs for expanded newborn screening have been implemented in Turkey (85) and Malaysia (142). Some progress in government support and expansion of neonatal screening programs has recently been achieved in India (143). Training in genetic counseling has been expanding in Asia and Africa (72).

An expanded neonatal program requires a developed infrastructure for interpreting findings, reporting, treatment, and counseling (43). Well-organized logistics of the screening program, from the screening laboratory to central clinical management, are essential (31). This may be why there is little information about newborn screening efforts in Nepal, Cambodia, Laos, and Pacific Island countries, and no organized screening efforts are reported from there. As approximately half of the world's births occur in the Asia-Pacific region, it is necessary to continue ongoing efforts to introduce and expand screening programs there so that children can achieve the same health status as children in more developed parts of the world (144).

A series of selective screening programs have been implemented in Egypt (76, 77), Saudi Arabia (84), and Morocco (145). In countries with high rates of consanguineous marriage, the incidence of many IEMs is significantly higher than in countries without such a problem (70, 77, 85). Pilot studies performed in some Middle Eastern countries show that the incidence of inborn metabolic disorders is higher in the region than anywhere else in the world due to consanguinity. This problem is relevant in Bahrain (71), Turkey (21, 85), Egypt (76, 77), Saudi Arabia (84), Lebanon (51), India (141, 143), and Oman (70). Using authors’ and additional keywords in visualization maps confirms a relatively high frequency of the terms “consanguinity” (Supplementary Figure 11B—orange cluster) and “selective screening” (Supplementary Figure 11A—dark blue cluster, Supplementary Figure 11B—orange cluster). The co-location of these two terms on the additional keyword map in the same cluster and close to each other indicates that they are often used together (Supplementary Figure 11B).

Analysis of authors’ and additional keywords, as well as keywords extracted from the articles’ abstracts and titles included in the current study, indicates their multiplicity, which is associated with both the wide range of IEMs and the rarity of IEMs in general (Supplementary Figures 9A,B, 11A,B). Keywords show changes in frequency of use associated with time trends (Supplementary Figures 9C,D, 10A,B), which is related to changes in the relevance of individual keywords, reflecting different directions in using MS/MS as an IEM screening tool over various periods.

One of the current trends in IEM is the development of new or improved diagnostic and treatment methods. The clinical effectiveness of MS/MS screening is unquestionable in some conditions but absent in others. The assessment of rarer diseases is more complex (19, 146). Next-generation sequencing in the form of whole exome and whole genome analysis is now strongly proposed as a potential alternative to mass spectrometric screening of newborns for IEM (19, 147–149). These methods have the advantages of high throughput, high accuracy, and the potential ability to detect all types of genetic disorders, even beyond IEMs, with almost equal sensitivity and specificity. However, major limiting factors include data interpretation dilemmas and the relatively high cost of such methods. As an alternative, a combination of MS/MS and sequencing is proposed (123, 125, 136, 150, 151). This trend is reflected in the increasing frequency of use of the term “next-generation sequencing,” as shown by visualization maps of the occurrence of keywords (Supplementary Figure 11A—purple cluster, Supplementary Figure 11B—red cluster), most frequent authors’ keywords (Supplementary Figure 9A), a graph of the evolution of authors’ keywords (Supplementary Figure 10B). Besides, the frequency of use the term “molecular genetics” in publications on MS/MS in IEM screening, has increased in recent years (Supplementary Figure 10A).

The high frequency of the term “false-positive rate” (Supplementary Figures 11A,B yellow cluster, Supplementary Figure 11C—red cluster) indicates the need to reduce false-positive results and avoid false-negative results associated with the development of optimal second-tier testing strategies. Studies of neonatal screening results present different rates of false-positive and false-negative results, probably due to differences in the approaches to the selection and use of second-tier tests. In the early stage of MS/MS use in IEM screening, false-positive results were higher. MS/MS-based newborn screening has shown specificity from 83% to 99% depending on the IEM (43). In a study by Schulze et al., the results of which were presented in 2003, a total of 0.33% of tests were false positive (44). La Marca et al. describe methods to reduce false results in MS/MS screening of newborns to 0.32%, with the introduction of second-tier tests (111). In the study by Vilarinho et al., 0.12% of tests were classified as false positives (114). Development of MS/MS-based second-level screening tests at Mayo Clinic reduces false positives to 0.09% (152). Postanalytical interpretive tools can identify false-positive IEM screening results (153, 154). Thus, using R4S (later CLIR) tools reduces the actual false positive rate from 0.26% to 0.02% (154). In Minnesota, programming tools have been a major factor in the steady decline in false positive testing rates below 0.1% (155). According to the results of the NBS conducted in Denmark, the frequency of false positive results was 0.038% (31). Neonatal screening results in Iran have found a false positive rate of 0.15% (156).

Shen et al.'s study showed a false positive rate of 1.4% in MS/MS screening compared with NGS. NGS enables rapid scanning of large gene panels and significantly improves the accuracy of NBS diagnosis. The screening efficiency using NGS is enhanced by expanding the spectrum of diseases (157). Lin et al. found that 24% (6/25) of PCD (primary carnitine deficiency) cases detected by second-level genetic testing would have been missed by conventional NBS (158). However, there are problems associated with the use of NGS in NBS, including the large number of variants with uncertain significance (VUS) and the lack of a recognized and recommended list of conditions associated with genes and diseases (159).

One option for using MS/MS for IEM selective screening is the retrospective analysis of dry blood spots (DBS) stored after neonatal screening. This option is used in developing countries with financial constraints where expanded newborn screening using MS/MS is impossible. In this regard, the issue of dry blood spot storage conditions is being studied. Among the papers on this topic are those from Asia because storage conditions for DBS in hot and humid climates are critical (143, 146, 160). However, the issue of storage conditions for DBS and the development of references and correction factors for metabolites is relevant not only to developing countries. Recent trends in creating biobanks and using stored samples for metabolomic studies, including disease prediction and understanding the basic molecular mechanisms of disease development, raise this issue for all researchers (161–163).

Thus, different regions and countries are currently at entirely different stages regarding using MS/MS in neonatal IEM screening. Some countries do not provide any data on screening for IEM, as research in this direction has not been carried out (Figure 3A). Developed countries have undergone pilot and experimental studies, and neonatal screening using MS/MS is now part of their national health programs.

Expanding screening programs has resulted in high heterogeneity in the IEMs included in different ENBS programs. In this regard, competent organizations in the USA and the European Union have proposed two unified screening panels (164). Currently, attention in developed countries is focused on the special considerations and limitations of newborn screening in sick and premature infants and some of the ethical issues associated with newborn screening. New disorders being considered for testing and new technologies that may be used for newborn screening are also discussed (125, 165, 166). New therapeutic modalities, such as enzyme replacement therapy and substrate reduction therapy, are being developed for many inborn errors of metabolism (19).

Some countries are at the stage of introducing MS/MS screening into public health programs. Some low-income countries have piloted expanded newborn screening programs or sporadic selective screening programs for IEM. In Latin America (105, 167–171), some countries in Africa (76, 77, 145), the Middle East (71, 84, 85), and the Asia-Pacific region (24, 142, 144, 172), there are some pockets of activity where new NBS programs are designed by partnerships between governments, non-governmental organizations, academia, the private sector, and civil society (173).

The analysis of the conceptual structure of the sample of 677 articles revealed several issues related to IEM screening and the use of MS/MS as a diagnostic tool.

1. Currently, newborn screening for IEM is unavailable in some countries due to limited access to advanced technologies for diagnosing and treating different types of IEM.
2. Most of the positive IEM screening results obtained with MS/MS are false positive, and second-level tests will be required.
3. In newborn screening, false negative results can have disastrous consequences, leading to disability and death. False positive results contribute to parental anxiety, unnecessary supervision, and financial costs.
4. Some IEMs are difficult to diagnose, have multiple clinical subtypes, and are not always treatable. Therapies are not available for all IEMs or are often expensive, risky, and of uncertain efficacy. This explains the careful evaluation of the possibilities of including new IEMs in screening panels.
5. Although the use of DBS is the gold standard for MS/MS screening for IEMs, it carries certain risks during storage and transportation, especially in regions with high temperatures and humidity.

Based on keyword analysis and co-citation, current trends and future areas of interest have been identified in the MS/MS newborn screening for IEMs.

1. Reducing the cost and improving the availability of diagnostic tests for IEMs, especially in resource-limited countries, is one of the most important public health challenges worldwide.
2. Further multiplexing of MS/MS tests would reduce the financial burden on public health and improve the efficiency of both first- and second-level screening.
3. Development of new mass spectrometry techniques that provide higher screening performance, such as ultra-high performance liquid chromatography-tandem mass spectrometry (UHPLC-MS/MS) and high-throughput tandem mass spectrometry (HT-MS/MS).
4. Reducing the level of false positive results through the use of second tier tests. Avoiding false negative results by using new specific biomarkers.
5. The development of metabolomic and proteomic approaches will lead to the application of new biomarkers for IEM.
6. Adding new treatable conditions to NBS programs.
7. Increasing the number of projects that use NGS significantly expands the range and capabilities of screening.

The performed bibliographic analysis highlighted substantial unevenness in the development of screening programs based on MS/MS on a global scale. However, the data obtained made it possible to identify the main directions for future screening technologies to detect inherited metabolic diseases. These positive aspects of the study can be referred to as undoubtful advantages. To our knowledge, we are the first researchers who tried to analyze the current bibliography on MS/MS neonatal screening at such a comprehensive level across countries, institutions, authors, journals, papers, and keywords.

Along with that, the study had inevitable limitations:

1. We used only the WoS Core Collection to search for relevant sources. The current study did not consider other databases, such as Scopus and MEDLINE. WoS is the most commonly used database in scientometrics, and Biblioshiny and VOSviewer have identified a format for recording metadata from WoS.
2. Only articles in English were included.
3. The study did not include proceeding papers, book chapters, meeting abstracts, editorial materials, early access articles, letters, and notes.
4. The total citation rate for newer articles is lower, which can be a manifestation of the bibliometric analysis's methodological weakness. However, this is covered by the total citation indicator per period (year)—TC per Year (Supplementary Table 1).
5. Bibliometric and scientometric analysis of articles indexed in the WoS database focused only on metadata, not their content. Analysis of the full text of the included articles and their scientific content was not the purpose of the research as being beyond the scope of this article. Besides, analyzing the textual content of the abstracts was also not the purpose of our study. Article metadata were sources of information about authors and their countries/institutions to assess their productivity, collaboration, and keyword trends.
6. The textual content of some images displayed by Biblioshiny and VOSviewer is incomplete.

## 5 Conclusions

This study was the first to explore trends in the use of MS/MS in IEM screening from 1991 to May 2024 through detailed bibliometric analysis. We identified publication and citation trends based on 677 articles retrieved from WoS. We analyzed the productivity and collaboration of countries, organizations, authors, and sources to determine the research status of MS/MS utilization for IEM screening. The research highlighted the crucial feature of using tandem mass spectrometry in identifying IEMs: the uneven involvement of countries in advanced newborn screening technologies due to their economic disparity.

Keywords and co-citation analysis identified the most relevant current research directions and future areas of interest. Based on the presented study, future research areas could include “screening for IEM in developing countries,” “selective screening for IEM,” “new treatments for IEM,” “new NBS programs,” “new disorders considered for MS/MS testing, “ethical issues related to newborn screening,” and “new technologies that may be used in the future for newborn screening.” In this relation, the “use of MS/MS and gene sequencing combination” is considered to be the most promising.
