# Supplementary material for: Tandem mass spectrometry in screening for inborn errors of metabolism: comprehensive bibliometric analysis
Source: Front Pediatr. 2025 Feb 20;13:1463294. doi: 10.3389/fped.2025.1463294 (PMC11882580; doi:10.3389/fped.2025.1463294)
Supplement: Supplementary file 12 [file Table1.docx]

**Supplementary Table 1:** Most cited articles.

| **Rank** | **Article Title** | **Journal** | **Year** | **First Author** | **Total Cita**  **tions** | **TC per Year** | **Norma-lized TC** | **Local Cita**  **tions** | **LC/TC Ratio (%)** | **Refe**  **rences** |
| --- | --- | --- | --- | --- | --- | --- | --- | --- | --- | --- |
| 1 | Screening newborn for inborn errors of metabolism by tandem mass spectrometry | New England Journal of Medicine | 2003 | Wilcken, B | 495 | 22.50 | 4.68 | 150 | 30.30 | [1] |
| 2 | Dried blood spot sampling in combination with LC-MS/MS for quantitative analysis of small molecules | Biomedical Chromatography | 2010 | Li, W | 477 | 31.80 | 8.52 | 7 | 1.47 | [2] |
| 3 | Tandem mass  spectrometric analysis for amino, organic, and fatty acid disorders in newborn dried blood spots: A two-year summary from the New England newborn  screening program | Clinical Chemistry | 2001 | Zytkovicz, TH | 400 | 16.67 | 3.67 | 101 | 25.25 | [3] |
| 4 | Expanded newbornscreening for inbor errors of metabolismby electrospray ionization-tandem mass spectrometry: Results, outcome, and implications | Pediatrics | 2003 | Schulze, A | 365 | 16.59 | 3.45 | 127 | 34.79 | [4] |
| 5 | Current status ofnewborn screeningworldwide: 2015 | Seminars in Perinatology | 2015 | Therrell, BL | 363 | 36.30 | 10.23 | 43 | 11.85 | [5] |
| 6 | Diagnosis ofinborn errorsof metabolismfrom blood spots by acylcarnitines and amino acids profiling using automated electrospraytandem mass spectrometry | Pediatric Research | 1995 | Rashed, MS | 281 | 9.37 | 1.95 | 69 | 24.56 | [6] |
| 7 | Clinical validationof cutoff target ranges in newbornscreening ofmetabolic disorders by tandem massspectrometry: A worldwide collaborative project | Genetics in Medicine | 2011 | McHugh, DMS | 265 | 18.93 | 5.89 | 63 | 23.77 | [7] |
| 8 | Effect of expandednewborn screeningfor biochemical genetic disorders on child outcomes and parental stress | Jama - Journal of the American Medical Assoc. | 2003 | Waisbren, SE | 258 | 11.73 | 2.44 | 43 | 16.67 | [8] |
| 9 | Rapid diagnosis ofMCAD deficiency: quantitative analysis ofoctanoylcarnitine and other acylcarnitines in newborn blood spots by tandemmass spectrometry | Clinical Chemistry | 1997 | Chace, DH | 236 | 8.43 | 1.46 | 48 | 20.34 | [9] |
| 10 | Screening blood spots for inbornerrors of metabolism by electrospraytandem massspectrometrywith a microplate batch process and a computer algorithm for automated flagging ofabnormal profiles | Clinical Chemistry | 1997 | Rashed, MS | 231 | 8.25 | 1.43 | 50 | 21.65 | [10] |
| 11 | Diagnosis and management of glutaric aciduria type I – revised recommendations | Journal of Inherited Metabolic Disease | 2011 | Kölker, S | 216 | 15.43 | 4.80 | 8 | 3.70 | [11] |
| 12 | Neonatal screeningfor lysosomal storage disorders: feasibility and incidence from a nationwide study in Austria | Lancet | 2012 | Mechtler, TP | 213 | 16.38 | 6.97 | 8 | 3.90 | [12] |
| 13 | Natural history, outcome, and treatment efficacy in children and adults with glutaryl-CoA dehydrogenase deficiency | Pediatric Research | 2006 | Kölker, S | 200 | 10.53 | 2.69 | 16 | 8.0 | [13] |
| 14 | Electrospraytandem massspectrometryfor analysis ofacylcarnitines in dried postmortem blood specimens collected at autopsy from infants with unexplained causeof death | Clinical Chemistry | 2001 | Chace, DH | 192 | 8.00 | 1.76 | 56 | 29.17 | [14] |
| 15 | Disorders ofmitochondrial long-chain fatty acid oxidation and the carnitine shuttle | Reviews in Endocrine & Metabolic Disorders | 2018 | Knottnerus, SJG | 182 | 26.00 | 6.15 | 2 | 1.10 | [15] |

References

1. Wilcken B, Wiley V, Hammond J, Carpenter K. Screening newborns for inborn errors of metabolism by tandem mass spectrometry. *N Engl J Med.* (2003) 348(23):2304-12. doi: 10.1056/NEJMoa025225.

2. Li W, Tse FL. Dried blood spot sampling in combination with LC-MS/MS for quantitative analysis of small molecules. *Biomed Chromatogr.* (2010) 24(1):49-65. doi: 10.1002/bmc.1367.

3. Zytkovicz TH, Fitzgerald EF, Marsden D, Larson CA, Shih VE, Johnson DM, et al. Tandem mass spectrometric analysis for amino, organic, and fatty acid disorders in newborn dried blood spots: a two-year summary from the New England Newborn Screening Program. *Clin Chem.* (2001) 47(11):1945-55.

4. Schulze A, Lindner M, Kohlmüller D, Olgemöller K, Mayatepek E, Hoffmann GF. Expanded newborn screening for inborn errors of metabolism by electrospray ionization-tandem mass spectrometry: results, outcome, and implications. *Pediatrics.* (2003) 111(6 Pt 1):1399-406. doi: 10.1542/peds.111.6.1399.

5. Therrell BL, Padilla CD, Loeber JG, Kneisser I, Saadallah A, Borrajo GJ, Adams J. Current status of newborn screening worldwide: 2015. *Semin Perinatol.* (2015) 39(3):171-87. doi: 10.1053/j.semperi.2015.03.002.

6. Rashed, M.S.; Özand, P.T.; Bucknall, M.P.; Little, D. Diagnosis of inborn errors of metabolism from blood spots by acylcarnitines and amino acids profiling using automated electrospray tandem mass spectrometry. *Pediatr Res.* 1995, *38(3),* 324-331. doi: 10.1203/00006450-199509000-00009.

7. McHugh D, Cameron CA, Abdenur JE, Abdulrahman M, Adair O, Al Nuaimi SA, et al. Clinical validation of cutoff target ranges in newborn screening of metabolic disorders by tandem mass spectrometry: a worldwide collaborative project. *Genet Med.* (2011) 13(3):230-54. doi: 10.1097/GIM.0b013e31820d5e67.

8. Waisbren SE, Albers S, Amato S, Ampola M, Brewster TG, Demmer L, et al. Effect of expanded newborn screening for biochemical genetic disorders on child outcomes and parental stress. *JAMA.* (2003) 290(19):2564-72. doi: 10.1001/jama.290.19.2564.

9. Chace DH, Hillman SL, Van Hove JL, Naylor EW. Rapid diagnosis of MCAD deficiency: quantitative analysis of octanoylcarnitine and other acylcarnitines in newborn blood spots by tandem mass spectrometry. *Clin Chem.* (1997) 43(11):2106-13.

10. Rashed MS, Bucknall MP, Little D, Awad A, Jacob M, Alamoudi M, et al. Screening blood spots for inborn errors of metabolism by electrospray tandem mass spectrometry with a microplate batch process and a computer algorithm for automated flagging of abnormal profiles. *Clin Chem.* (1997) 43(7):1129-41.

11. Kölker S, Christensen E, Leonard JV, Greenberg CR, Boneh A, Burlina AB, et al. Diagnosis and management of glutaric aciduria type I--revised recommendations. *J Inherit Metab Dis.* (2011) 34(3):677-94. doi: 10.1007/s10545-011-9289-5.

12. Mechtler TP, Stary S, Metz TF, De Jesús VR, Greber-Platzer S, Pollak A, Herkner KR, Streubel B, Kasper DC. Neonatal screening for lysosomal storage disorders: feasibility and incidence from a nationwide study in Austria. *Lancet.* (2012) 379(9813):335-41. doi: 10.1016/S0140-6736(11)61266-X.

13. Kölker S, Garbade SF, Greenberg CR, Leonard JV, Saudubray JM, Ribes A, et al. Natural history, outcome, and treatment efficacy in children and adults with glutaryl-CoA dehydrogenase deficiency. *Pediatr Res.* (2006) 59(6):840-7. doi: 10.1203/01.pdr.0000219387.79887.86.

14. Chace DH, DiPerna JC, Mitchell BL, Sgroi B, Hofman LF, Naylor EW. Electrospray tandem mass spectrometry for analysis of acylcarnitines in dried postmortem blood specimens collected at autopsy from infants with unexplained cause of death. *Clin Chem.* (2001) 47(7):1166-82. Erratum in: *Clin Chem* (2001) 47(9):1748.

15. Knottnerus SJG, Bleeker JC, Wüst RCI, Ferdinandusse S, IJlst L, Wijburg FA, et al. Disorders of mitochondrial long-chain fatty acid oxidation and the carnitine shuttle. *Rev Endocr Metab Disord.* (2018) 19(1):93-106. doi: 10.1007/s11154-018-9448-1.
